# Validation of a Consensus Method for Identifying Delirium from Hospital Records

**DOI:** 10.1371/journal.pone.0111823

**Published:** 2014-11-04

**Authors:** Elvira Kuhn, Xinyi Du, Keith McGrath, Sarah Coveney, Niamh O'Regan, Sarah Richardson, Andrew Teodorczuk, Louise Allan, Dan Wilson, Sharon K. Inouye, Alasdair M. J. MacLullich, David Meagher, Carol Brayne, Suzanne Timmons, Daniel Davis

**Affiliations:** 1 Centre for Gerontology and Rehabilitation, School of Medicine, St. Finbarr's Hospital, Cork, Ireland; 2 School of Medicine, University of Cambridge, Cambridge, United Kingdom; 3 Institute of Neuroscience, Newcastle University, Newcastle, United Kingdom; 4 Department of Clinical Gerontology, Kings College Hospital NHS Foundation Trust, London, United Kingdom; 5 Aging Brain Center, Institute for Aging Research, Hebrew SeniorLife, and Department of Medicine, Beth Israel Deaconess Medical Center, Harvard Medical School, Boston, United States of America; 6 Centre for Cognitive Ageing and Cognitive Epidemiology, University of Edinburgh, Edinburgh, United Kingdom; 7 Edinburgh Delirium Research Group, University of Edinburgh, Edinburgh, United Kingdom; 8 Department of Psychiatry, University of Limerick, Limerick, Ireland; 9 Department of Public Health and Primary Care, University of Cambridge, Cambridge, United Kingdom; 10 MRC Unit for Lifelong Health and Ageing, University College London, London, United Kingdom; Cardiff University, United Kingdom

## Abstract

**Background:**

Delirium is increasingly considered to be an important determinant of trajectories of cognitive decline. Therefore, analyses of existing cohort studies measuring cognitive outcomes could benefit from methods to ascertain a retrospective delirium diagnosis. This study aimed to develop and validate such a method for delirium detection using routine medical records in UK and Ireland.

**Methods:**

A point prevalence study of delirium provided the reference-standard ratings for delirium diagnosis. Blinded to study results, clinical vignettes were compiled from participants' medical records in a standardised manner, describing any relevant delirium symptoms recorded in the whole case record for the period leading up to case-ascertainment. An expert panel rated each vignette as unlikely, possible, or probable delirium and disagreements were resolved by consensus.

**Results:**

From 95 case records, 424 vignettes were abstracted by 5 trained clinicians. There were 29 delirium cases according to the reference standard. Median age of subjects was 76.6 years (interquartile range 54.6 to 82.5). Against the original study DSM-IV diagnosis, the chart abstraction method gave a positive likelihood ratio (LR) of 7.8 (95% CI 5.7–12.0) and the negative LR of 0.45 (95% CI 0.40–0.47) for probable delirium (sensitivity 0.58 (95% CI 0.53–0.62); specificity 0.93 (95% CI 0.90–0.95); AUC 0.86 (95% CI 0.82–0.89)). The method diagnosed possible delirium with positive LR 3.5 (95% CI 2.9–4.3) and negative LR 0.15 (95% CI 0.11–0.21) (sensitivity 0.89 (95% CI 0.85–0.91); specificity 0.75 (95% CI 0.71–0.79); AUC 0.86 (95% CI 0.80–0.89)).

**Conclusions:**

This chart abstraction method can retrospectively diagnose delirium in hospitalised patients with good accuracy. This has potential for retrospectively identifying delirium in cohort studies where routine medical records are available. This example of record linkage between hospitalisations and epidemiological data may lead to further insights into the inter-relationship between acute illness, as an exposure, for a range of chronic health outcomes.

## Introduction

Delirium is a severe, acute neuropsychiatric syndrome which affects at least 1 in 8 hospital inpatients [1]. It is associated multiple adverse outcomes, including increased risk of complications, longer length of stay, mortality, and high levels of personal and family distress [2]–[4]. Delirium is also associated with enormous healthcare costs, with UK analyses estimating an extra £13,200 per hospital admission [5]. It is characterised by an acute and fluctuating impairment of cognition and attention precipitated by medical illness. It mainly affects older adults, especially those with pre-existing cognitive impairment and other comorbidities.

It is well recognised that delirium during hospitalisation is associated with poor cognitive outcomes [2]. Indeed, because delirium is partly preventable [6], [7], delirium interventions might even prevent dementia [8], [9]. However, around half of dementia presenting to hospital is undiagnosed [10], and there is often uncertainty about an individual's premorbid cognitive function. Accordingly, hospital series may overestimate the association between delirium and any subsequent cognitive impairment.

The prospective relationship between delirium and dementia is more reliably assessed by ascertaining incident delirium in the context of a cohort study measuring cognitive outcomes. Nonetheless, only one prospective study has specifically examined cognitive outcomes after delirium in the general population [11], [12]. Given the wider importance of delirium's association with dementia, attempts to identify delirium in other cohort studies would be highly informative, even if the delirium measures were retrospectively derived.

Delirium is under-diagnosed and under-reported such that medical records are known to be unreliable sources for delirium [13]. Despite this, a chart-based method for retrospectively identifying delirium has been validated against trained interviewers using the Confusion Assessment Method (CAM) as a reference standard [14], [15]. This instrument has been innovative in identifying incident delirium in community-based persons with dementia being followed up with regular cognitive assessments, showing an association with more rapid trajectories of decline [16], [17]. This tool was developed in the US healthcare system and there are differences in how medical records are kept in the UK and Ireland. Hence there may be a need for a complementary approach for use outside the USA.

The aim of the present study is to develop and validate a retrospective measure of delirium based on routine medical records used in the general hospital setting in the UK and Ireland. From the medical records of participants in an independent study of delirium prevalence [18], two separate processes were employed: (i) abstraction of symptoms relevant to the Diagnostic and Statistical Manual of Mental Disorders (Fourth Edition) (DSM-IV) criteria for delirium [19] to produce a short clinical vignette; (ii) an expert panel assigning diagnoses by consensus (index test). These diagnoses could then be validated against the DSM-IV diagnosis of delirium (reference standard) applied as part of the delirium prevalence study.

## Methods

The protocol followed the STAndards for the Reporting of Diagnostic accuracy studies (STARD) guidelines [20].

### Delirium reference standard

The reference standard for delirium was based on direct clinical assessment in the Cork Delirium Point Prevalence Study [18]. In this study, the entire adult inpatient population of a general hospital (excluding ICU and moribund patients) was examined for delirium over a single day. Assessments were performed in two stages. Firstly, participants were screened for inattention using the spatial span forwards (where participants are asked to remember the sequence of coloured dots presented on a card) and months backwards. Participants were additionally screened for subjective confusion by asking: “Have you felt muddled in your thinking, or confused, since you came into hospital?” Further information was derived from nurse informants and hospital records. Those screening positive on any of these components, and a random sample of screen negative participants, were assessed in more detail. The second stage consisted of two independent delirium assessments: the CAM [15] and the Delirium Rating Scale – Revised-98 (DRS-R98) [21]. These were conducted by trained registrars or consultants in geriatric medicine and experienced psychiatrists. The final diagnosis of delirium was based on DSM-IV criteria, applied by consensus using all available psychometric, clinical and informant data. Accordingly, all persons in the prevalence study were thus assigned a diagnosis of ‘delirium’ or ‘no delirium’ for a specific day.

### Dementia status

Evidence for pre-existing cognitive impairment or dementia (e.g. diagnosis made at a memory clinic) was sought through examination of the medical notes. If this was not apparent, premorbid cognition was assessed using the short form of the Informant Questionnaire on Cognitive Decline in the Elderly (IQCODE) [22]. This was done for all participants with delirium (n = 55) as well as a random subsample of those aged ≥65 years without delirium (n = 40).

### Chart abstraction technique

A random selection of case notes was identified using the RAND() function in Excel. The sample was designed to maintain the underlying prevalence of delirium (that is, 20% of the identified hospital records were delirium cases). The case notes were then requested from the medical records department on a convenience basis, in batches.

All relevant clinical documentation was scanned for keywords (Table 1) and used for abstraction. This included all entries by medical, nursing, therapy and social work staff from the date of admission, up until the date of the point-prevalence study (15/05/2010). If the inpatient stay had been longer than two weeks, only clinical information from the two weeks leading up to the index date was used. This included verbatim reports from the entirety of the medical, nursing and allied health professional records.

**Table 1:** Abstracted symptoms in relation to DSM-IV criteria.

DSM-IV criterion	Abstracted symptoms
A. Disturbance of consciousness (i.e., reduced clarity of awareness of the environment) with reduced ability to focus, sustain or shift attention.	Agitation; drowsiness; any formal rating e.g. AVPU or GCS. Any verbatim comment, e.g. ‘drowsy’, ‘slept poorly’, ‘agitated’
B. A change in cognition or the development of a perceptual disturbance that is not better accounted for by a pre-existing, established or evolving dementia.	Any formal cognitive assessment (AMT; MMSE) Any formal specialty assessment, e.g. neurology, geriatric medicine, liaison psychiatry. Any verbatim comment, e.g. ‘more confused’, ‘disorientated’
C. The disturbance develops over a short period of time (usually hours to days) and tends to fluctuate during the course of the day	Observations at least three times daily (nursing). Any verbatim comment indicating change in mental state.
D. There is evidence from the history, physical examination or laboratory findings that the disturbance is caused by the direct physiological consequences of a general medical condition.	General clinical vignette, including metabolic and laboratory parameters taken closest to date of prevalence study: AVPU score; systolic blood pressure; pulse; respiratory rate; oxygen saturation; temperature; C-reactive protein; urea; creatinine.

AVPU = assessment of arousal where categories are Alert, Verbally-responsive, Pain-responsive, Unresponsive.

GCS = Glasgow Coma Scale.

AMT = Abbreviated Mental Test.

MMSE = Mini-Mental State Examination.

Evidence for each criterion of the DSM-IV classification was sought, along with specific synonyms or clinically recorded parameters (Table 1). For example, evidence for Criterion A (disturbance of consciousness) might include references to agitation, drowsiness, or any formal rating of arousal (e.g. Alert-Voice-Pain-Unresponsive). All verbatim comments, e.g. “drowsy”, “agitated”, were recorded for each Criterion A-D, resulting in a clinical vignette (see Supporting Information: File S1 for typical examples, fictionalised for confidentiality). The Charlson co-morbidities index [23], metabolic and physiological parameters were recorded closest to the date the reference standard was assessed.

Abstractors were specialist trainees in geriatric medicine, that is, qualified physicians undergoing postgraduate training in geriatric medicine. Each received a half-day training session and the first five abstractions were performed together. Time taken to produce a vignette was not formally recorded, but could take between 5 and 30 minutes, depending on the complexity of the inpatient episode. Case notes were abstracted multiple times to assess the influence of abstracting author on the consensus process. The inter-rater reliability was therefore tested by separately submitting each vignette to the consensus panel and then assessing if vignette abstractor was associated with changes in final diagnostic outcome.

### Consensus diagnosis

The consensus diagnosis process was the basis of the index test. The consensus panel comprised three geriatricians and an old age psychiatrist, all of whom provide specialist clinical services for delirium patients (LA, AT, DW, AMacL). Assessors only had access to the abstracted vignettes, and rated each independently as unlikely, possible or probable delirium. Assessors were asked to use each criterion from the DSM-IV classification to support their assigned diagnoses. Cases where the initial diagnoses were not unanimous were re-examined together until consensus was reached.

### Statistical methods

All analyses were conducted in Stata, version 12.1 (Stata Corps, Texas, USA). Sensitivities, specificities, positive and negative likelihood ratios were calculated from 2×2 tables, with confidence intervals testing significance at 95%. ROC curves were derived from estimates of sensitivity and specificity. For each individual with multiple vignettes (one vignette per abstractor), Fisher's exact test was used to assess if differences in the initially-assigned diagnostic categories varied according to abstractor.

### Ethical procedures

In the original study, the Research Ethics Committee, University College Cork approved the use of patient assent, augmented by written proxy consent. This included examination of the medical records. Approval for the present study using secondary data was approved by the same committee (ECM4(e)12/06/12). Additional written consent was not sought from the original participants, but all vignettes were anonymised and de-identified prior to analysis.

## Results

**Figure 1:**
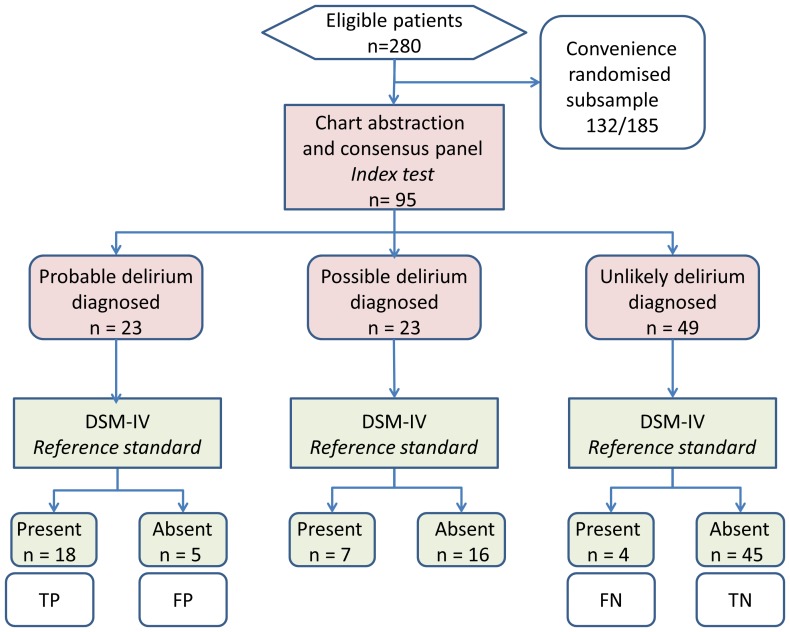
STARD flow diagram showing the numbers receiving the index test and reference standard. TP true positive; TN true negative; FP false positive; FN false negative.

Case records from 95 individuals were retrieved (Figure 1). Two or more abstractors (EK, KMcG, SC, NO'R, DD) separately extracted 424 independent vignettes. The characteristics of participants are summarised in Table 2. Median age was 76.6 years (interquartile range 54.6 to 82.5 years), 49% were women (n = 47), and median co-morbidity score was 3 (interquartile range 1 to 5). Dementia status was ascertained as part of the point prevalence study in 31 persons (target subsample aged ≥65+ all delirium cases), with a prevalence of 10/31 (32%). Table 2 describes physiological (level of consciousness, heart rate, respiratory rate, systolic blood pressure, temperature, oxygen saturation, inspired oxygen) and metabolic (C-reactive protein, urea: creatinine ratio) characteristics in those with and without delirium. No significant differences were found, except that all non-delirious participants were ‘alert’ on the AVPU scale (arousal scale where categories are ‘alert’, ‘verbally responsive’, ‘pain responsive’ and ‘unresponsive’), compared with 3 participants with delirium being less than alert (p = 0.03).

**Table 2:** Characteristics of participants, by delirium status.

	DSM delirium (n = 29)	No DSM delirium (n = 66)	P value*
Age	80.6 (74.9–88.6)	68.2 (53.5–80.2)	0.07
Sex	14 (50%)	33 (50%)	1.00
Dementia	6/9	3/22	0.01
Co-morbidity score	4 (2–6)	2 (0–4)	0.44
Median CRP	57.3 (13–121.2)	37.0 (0—120.0)	0.49
Median Urea∶ creatinine	0.11 (0.09–0.14)	0.08 (0.06–0.10)	0.45
Median ViEWS			
AVPU	A = 26/29 V/P/U = 3/29	A = 66/66	0.03
HR	82.0	82.3	0.99
RR	19.5	18.7	0.31
BP	125	124	0.99
Temp	36.2	36.6	0.10
Sa0_2_	96	96	0.99
Fi0_2_	Y = 6 N = 23	Y = 9 N = 57	0.38

DSM delirium = reference standard delirium.

Dementia ascertained through IQCODE.

Co-morbidity score = Charlson co-morbidity index.

CRP C-reactive protein.

AVPU = assessment of arousal where categories are Alert, Verbally-responsive, Pain-responsive, Unresponsive.

Fi0_2_ is scored as Y = supplemental oxygen; N = room air.

*Aggregate information derived from multiple vignettes, therefore the standard errors (not shown) are not robust to the clustered nature of the data. The p values *are* derived from estimates with robust standard errors.

Table 3 gives the diagnostic test accuracy of the individual expert raters. Using a cut-point for ‘possible delirium’, ratings performed by experts individually (prior to consensus panel meeting) demonstrated sensitivity of 0.84 and specificity of 0.77. At a higher threshold for ‘probable delirium’, sensitivity was 0.63 and specificity 0.92 (AUC 0.84, 95% confidence interval (CI) 0.80 to 0.89). Furthermore, the individual DSM-IV criteria performed less well than the panel's overall impression (Table 3). Insofar as these could be evidenced in the clinical record, the order of test accuracy for each criterion (highest to lowest) was: change in cognition (B), demonstration of an acute change (C), documentation of inattention (A), physiological precipitant (D).

**Table 3:** Diagnostic test accuracy of the consensus method for delirium detection.

	Sensitivity			Specificity			LR+			LR−			AUROC		
		LCI	UCI		LCI	UCI		LCI	UCI		LCI	UCI		LCI	UCI
**DSM-IV Criteria**															
Inattention	0.68	0.63	0.72	0.86	0.83	0.89	4.83	3.59	6.58	0.38	0.32	0.45	0.77	0.73	0.81
Change in cognition	0.71	0.67	0.75	0.92	0.89	0.94	9.14	6.21	13.41	0.31	0.26	0.38	0.82	0.78	0.85
Acute and fluctuating	0.70	0.66	0.74	0.89	0.85	0.91	6.16	4.48	8.65	0.34	0.28	0.40	0.79	0.75	0.83
Physiological precipitant	0.68	0.63	0.72	0.82	0.78	0.86	3.83	2.91	5.01	0.39	0.33	0.47	0.75	0.71	0.79
**Possible delirium**															
Before consensus	0.84	0.80	0.87	0.77	0.72	0.80	3.62	2.91	4.46	0.21	0.16	0.27	0.85	0.81	0.88
Final consensus	0.89	0.85	0.91	0.75	0.71	0.79	3.54	2.90	4.32	0.15	0.11	0.21	0.86	0.82	0.89
Subgroup aged ≥70 years	0.88	0.83	0.92	0.68	0.62	0.73	2.74	2.17	3.43	0.18	0.12	0.27	0.82	0.77	0.87
Subgroup with dementia	0.88	0.69	0.96	0.57	0.39	0.76	2.06	1.13	3.90	0.21	0.06	0.80	0.69	0.51	0.85
**Probable delirium**															
Before consensus	0.63	0.58	0.67	0.92	0.89	0.94	7.97	5.30	11.62	0.40	0.35	0.47	0.85	0.81	0.88
Final consensus	0.58	0.50	0.62	0.93	0.90	0.95	7.80	5.65	11.96	0.46	0.40	0.47	0.86	0.82	0.89
Subgroup aged ≥70 years	0.54	0.48	0.60	0.90	0.85	0.93	5.33	3.23	8.49	0.51	0.43	0.61	0.82	0.77	0.87
Subgroup with dementia	0.71	0.51	0.85	0.57	0.39	0.76	1.65	0.83	3.47	0.51	0.20	1.27	0.69	0.51	0.85

LR Likelihood ratio; AUROC area under the curve, LCI lower 95% confidence interval; UCI upper 95% confidence interval.

After a consensus diagnosis was applied, there was a small improvement in diagnostic test accuracy. Vignette abstractor was not significantly associated with the eventual consensus diagnosis. For ‘possible delirium’, sensitivity was 0.88 and specificity 0.75; ‘probable delirium’ showed sensitivity 0.58 and specificity 0.93 (AUC 0.86, 95% CI 0.82 to 0.89). The positive likelihood ratio (LR) was 7.8 and the negative LR was 0.45. This indicates that cases deemed to be positive by the consensus panel were 7.8 times more likely to have delirium than not have delirium. With a delirium prevalence of 31%, the post-test probability of having ‘probable delirium’ given a positive chart identification is 82% (95% CI 73% to 89%).

In this sample, delirium was present in 50% of the participants aged ≥70 years. With LR+ = 5.3 and LR− = 0.5, the post-test probability for ‘probable delirium’ increased to 84% (95% CI 74% to 92%). Therefore, depending on the setting, the chart based abstraction method had a moderate impact on decision making.

Table 3 also shows that sensitivity for ‘possible delirium’ remains high (0.88) in the subgroup of persons aged ≥70 years (n = 57) (AUC 0.82, 95% CI 0.77 to 0.87). In the 31 persons with prior cognitive impairment identified by previously documented dementia or by IQCODE score (≥3.5), sensitivity for ‘possible delirium’ and ‘probable delirium’ was 0.88 and 0.71 respectively. Specificity in this group was 0.57 for both ‘possible delirium’ and ‘probable delirium’ (AUC 0.69, 95% CI 0.44 to 0.94).

Ten cases (11%) were retrieved for which no usable vignette could be abstracted, due to insufficient clinical records in the period leading up to the day of the point prevalence study. Whether a vignette could yield sufficient information was decided by consensus.

## Discussion

Here we present a new method for retrospectively ascertaining delirium from health care records, extending the original work in the US setting (see below) [14]. We found that diagnoses assigned by consensus panel based on abstracted clinical vignettes (index test) was sensitive to ‘possible delirium’ and more specific to ‘probable delirium’ when compared to DSM-IV diagnoses applied during assessment by a psychiatrist or geriatrician (reference standard). The diagnostic test accuracy remained similar in the subgroup of persons aged ≥70 years, though performed less well in the group with dementia. Overall, the likelihood ratios suggest that positive identification of probable delirium had a moderate effect on decision making.

One other approach has pioneered the use of medical records to derive a retrospective measure [14]. Developed in the US healthcare system, it has been effective in leveraging information from dementia cohorts. That study was larger, and used different methods. Firstly, a one-stage approach was used for abstraction and diagnosis (with variation in agreement assessed by kappa). Secondly, the CAM administered by expert assessors after cognitive testing was used as a reference standard, though this has very high sensitivity and specificity for DSM diagnoses in the centre developing the abstraction tool. As with our findings, diagnostic test accuracy was lower in the group with dementia, also ascertained through an informant test (modified Short Blessed Test [24]). The overall accuracy of the US chart technique reported sensitivity 0.74 and specificity 0.83. Our findings are comparable, though the outcome from the consensus panel in the present paper offered ‘possible’ (when sensitivity is more important), and ‘probable’ (when specificity is more important) diagnostic categories.

The strengths of our study lie in the use of routine clinical records of participants which were compared to expert delirium assessments. The consensus panel builds on a standard approach to case-ascertainment in psychiatric epidemiology. Use of multiple vignettes showed that the two-stage (diagnostic) process was robust, as variations between abstractors recorded in the vignette did not ultimately influence the diagnosis reached at consensus. That is, slight variations in abstracted information due to individual abstractors did not affect the overall judgements. It should be noted that abstractors in this study had more general clinical training than the method using nurse abstractors [14]. This may account for the greater inter-abstractor agreement shown here, rather than use of the consensus panel itself.

Certain limitations must also be acknowledged. The process was relatively time consuming, though we have established that multiple abstractions are not necessary. Diagnoses could not be assigned in 11% of cases due to insufficient data from routine clinical records. It is also possible that hypoactive delirium is under-recognised by this method. Finally, the consensus process for establishing diagnosis is not practical for routine clinical use, though may still have a role where delirium occurrence is a focus of service quality improvement evaluations.

The present results indicate that routine clinical data can be used to systematically gather information on delirium. In adapting this technique from the US model, we show that the same is achievable in UK/Irish systems, generally confirming the principle established by Inouye *et al.* [14] There is the potential to utilise the consensus approach to establish evidence of incident delirium during hospitalisation, improving standardisation of diagnostic categories. In addition, the technique might also have a place in clinical governance and audit. Future work should be directed towards use in existing and on-going studies where the relationship between delirium and adverse clinical outcomes are of interest. More broadly, there are general implications for the use of record linkage between acute hospitalisations and epidemiological data, where further insights on the inter-relationship between acute illness (as an exposure) and a range of chronic health outcomes.

## Supporting Information

File S1 **Four examples of abstracted case vignettes.** These are fictionalised given their clinical origins, but are typical cases.
(XLSX)
